# Supplementary material for: Towards unified AI-driven fracture mechanics: the extended deep energy method (XDEM)
Source: Nat Commun. 2026 Aug 15;17:8492. doi: 10.1038/s41467-026-76748-1 (PMC13484471; doi:10.1038/s41467-026-76748-1)
Supplement: Supplementary file 3 [file 41467_2026_76748_MOESM3_ESM.zip › pic/Appendix/UEL/Shape_function.pdf]

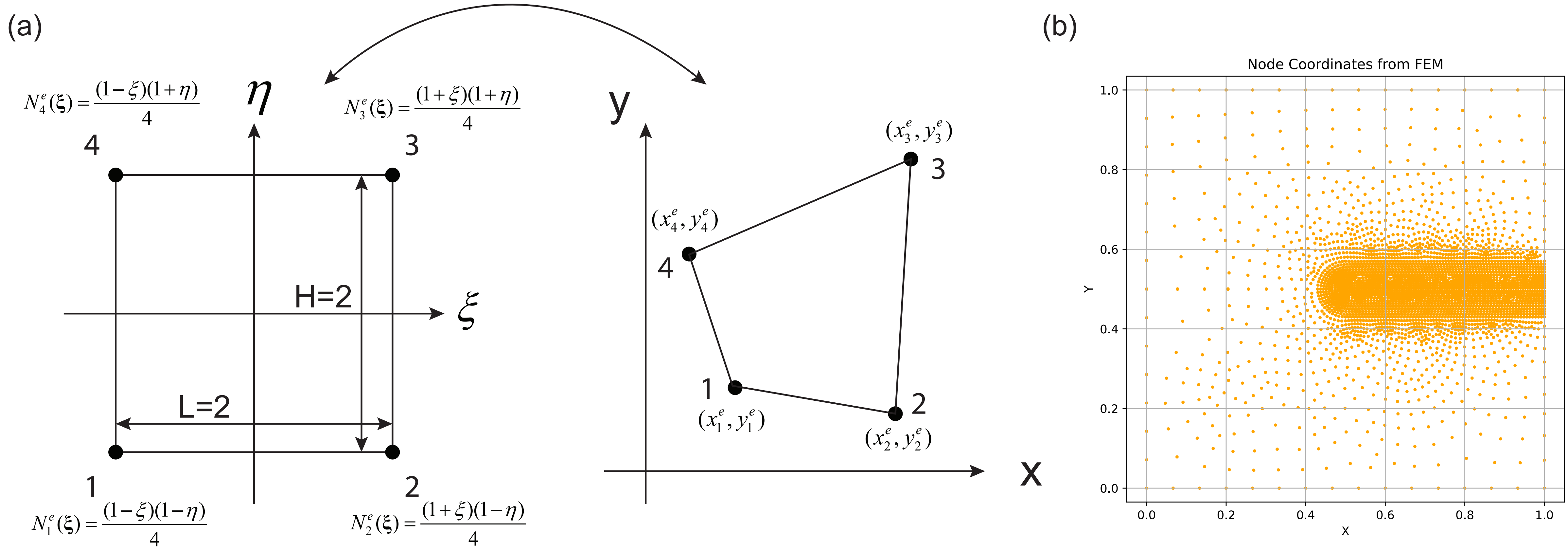
(a)
$N_4^e(\xi) = \frac{(1-\xi)(1+\eta)}{4}$
η
$N_3^e(\xi) = \frac{(1+\xi)(1+\eta)}{4}$
4
3
H=2
ξ
L=2
1
2
$N_1^e(\xi) = \frac{(1-\xi)(1-\eta)}{4}$
$N_2^e(\xi) = \frac{(1+\xi)(1-\eta)}{4}$
y
(x_3^e, y_3^e)
3
(x_4^e, y_4^e)
4
1
(x_1^e, y_1^e)
2
(x_2^e, y_2^e)
x
(b)
Node Coordinates from FEM
Y
X
0.0
0.2
0.4
0.6
0.8
1.0
